# The Essential Role of Prolines and Their Conformation in Allosteric Regulation of Kaiso Zinc Finger DNA-Binding Activity by the Adjacent C-Terminal Loop

**DOI:** 10.3390/ijms232415494

**Published:** 2022-12-07

**Authors:** Elena Belova, Oksana Maksimenko, Pavel Georgiev, Artem Bonchuk

**Affiliations:** 1 Department of the Control of Genetic Processes, Institute of Gene Biology Russian Academy of Sciences, 34/5 Vavilov St., Moscow 119334, Russia; 2 Center for Precision Genome Editing and Genetic Technologies for Biomedicine, Institute of Gene Biology, Russian Academy of Sciences, 34/5 Vavilov St., Moscow 119334, Russia

**Keywords:** DNA methylation, C2H2 zinc finger proteins, molecular dynamics, methyl(DNA)-binding proteins

## Abstract

Kaiso is a methyl-DNA-binding protein containing three C2H2 zinc fingers with a C-terminal extension that participates in DNA binding. The linker between the last zinc finger and the DNA-binding portion of the extension contains two prolines that are highly conserved in vertebrates and in cognate ZBTB4 and ZBTB38 proteins. Prolines provide chain rigidity and can exist in cis and trans conformations that can be switched by proline isomerases, affecting protein function. We found that substitution of the conserved proline P588, but not of P577, to alanine, negatively affected KaisoDNA-binding according to molecular dynamics simulation and in vitro DNA-binding assays. Molecular dynamics simulations of the Kaiso DNA-binding domain with P588 either substituted to alanine or switched to the cis-conformation revealed similar alterations in the H-bonding network and uncovered allosteric effects leading to structural rearrangements in the entire domain that resulted in the weakening of DNA-binding affinity. The substitution of proline with a large hydrophobic residue led to the same negative effects despite its ability to partially rescue the intrinsic DNA-binding activity of the C-terminal loop. Thus, the presence of the C-terminal extension and cis-conformation of proline residues are essential for efficient Kaiso–DNA binding, which likely involves intramolecular tension squeezing the DNA chain.

## 1. Introduction

Transcription factors with C2H2-type zinc-finger domains (C2H2 domains) form the largest protein family in bilateral organisms [1,2,3,4]. C2H2 domains have the consensus sequence CX2-4CX12HX2-8H, where X is any residue, and their structure is fastened by zinc coordinated with two cysteines at one end of the β-sheet and two histidines at the C-terminus of the α-helix [5]. In many transcription factors, C2H2 domains are clustered, determining highly specific binding to long DNA motifs. In the canonical complex, tandem C2H2 domains are embedded in the DNA major groove by α-helical regions [6,7]. Amino acids in the key positions of the α-helix specifically interact with the DNA triplet, which makes it possible to select a combination of C2H2 domains in the cluster to specifically recognize any DNA sequence [7]. Typically, C2H2 domains are separated by five amino acid linkers, which have a critical effect on the affinity and specificity of C2H2 cluster binding to DNA [8].

The linkers between individual zinc fingers often contain conserved proline [8]. Proline is unique among amino acids in its ability to adopt completely distinct cis and trans conformations [9]. It has previously been shown that the binding of some transcription factors to DNA can be regulated by cis–trans isomerization of proline, which is controlled by a specific class of enzymes called peptidyl-prolyl isomerases [10,11]. For example, the peptidyl-prolyl isomerase Pin1 promotes p53 and c-Myc DNA binding via the isomerization of prolines located outside of their DNA-binding domains [12,13].

**Figure 1:**
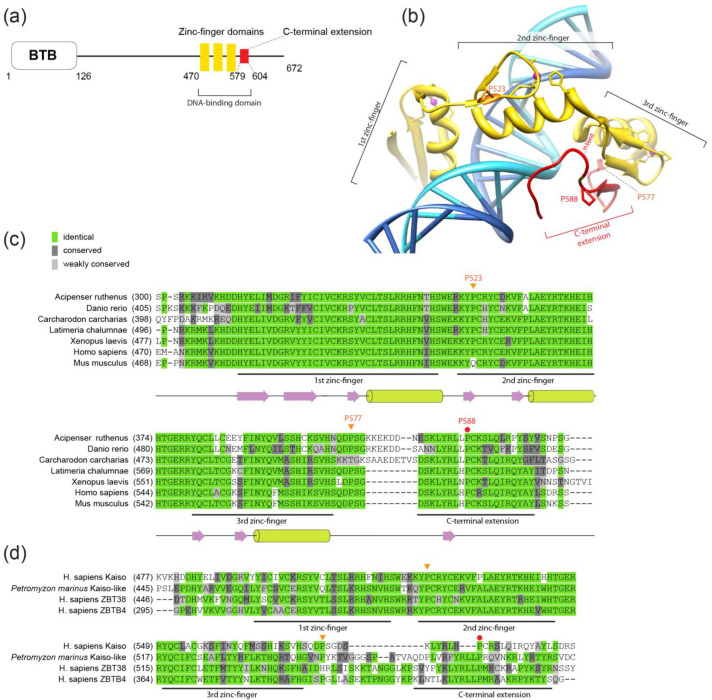
(**a**) Schematic representation of Kaiso domain structure, with the protein regions involved in DNA binding indicated. (**b**) Structure of Kaiso–DNA complex (PDB:4f6n [15]). (**c**) Multiple sequence alignment of the DNA-binding domains of Kaiso proteins from different vertebrates. P588 is indicated with a red circle, and P523 and P577 are indicated with orange triangles. (**d**) Multiple sequence alignment of DNA-binding domains from human Kaiso, ZBTB4, and ZBTB38 proteins and the Kaiso-like protein from the most ancient living vertebrate, *Petromyzon marinus* (lamprey). Molecular graphics were prepared using UCSF Chimera [30]. Multiple sequence alignments were prepared using the CLUSTALW algorithm [31].

The aim of this study is to assess the role of prolines and their conformation within the C-terminal loop adjacent to the zinc fingers of the human C2H2 protein Kaiso in its binding to DNA sites in vitro. Kaiso contains three C2H2 domains, for which the DNA-binding mechanism has been well studied (Figure 1a,b) [14,15]. Kaiso contains an N-terminal BTB/POZ domain and is a transcription factor that can stimulate or repress transcription depending on co-bound regulators [16,17,18]. It is also involved in the regulation of DNA methylation (methylation of cytosines at the 5 position) [19,20]. Abnormal Kaiso expression is frequently associated with cell malignancy [21,22,23,24,25,26]. Unlike most C2H2 proteins, which cannot bind effectively to methylated CpG, Kaiso binds to both the methylated repeat (CpG)2 and a longer unmethylated motif (TCCTGCNA) called the Kaiso binding sequence (KBS) [16,17,27]. Genome-wide studies performed on Caki-1 cells and other human cell lines have shown that most Kaiso binding sites are located in promoters of active genes containing CpG islands [19]. The first two C2H2 domains mediate contacts in the major groove with 5 base pairs in the core motif, while in the third C2H2 domain and the adjacent conserved C-terminal, 31 amino acids bind in the opposing minor groove and are required for high-affinity Kaiso binding to both the KBS and methylated CpG repeats [14,15,28]. The C-terminal region is disordered and adopts an ordered structure upon binding of Kaiso to DNA [15].

Kaiso is found in most vertebrates [29] and contains two highly conserved prolines (P577 and P588) that are located in the linker connecting the third zinc finger to the C-terminal region that enhances DNA binding. Here, we explored the significance of these prolines and their conformation for Kaiso DNA-binding activity using molecular dynamics (MD) simulations and in vitro DNA-binding assays with mutant derivatives of the Kaiso protein. We found that the rigidity provided by the trans-conformation of P588 was important for DNA binding and that DNA-binding activity may be regulated by a cis–trans switch mediated by proline-isomerase family proteins. Multiple allosteric effects of alterations in the C-terminal loop suggest the existence of a DNA-binding mechanism involving intramolecular tension.

## 2. Results

### 2.1. Kaiso Has Conserved Prolines in the C-Terminal Region That are Involved in DNA Binding

The three zinc-finger domain of Kaiso contains the 31-bp C-terminal region called the C-terminal extension (Figure 1a,b), which also contributes to DNA binding through interaction with the minor groove [15]. The first part of the C-terminal extension (aa 579–587) forms an additional beta-strand with the third zinc finger, while the second part (aa 592–604) participates in DNA binding by itself and forms a hydrogen bond (H-bond) with the second zinc-finger domain when the protein is bound to DNA (Figure 1b). The parts of the C-terminal extension are connected by the linker (aa 587–591), which includes the conserved proline P588 (Figure 1b,c). The whole C-terminal extension is disordered in solution [15] and is linked to the C2H2 domains by the short sequence that includes the proline residue P577.

Proline residues within a linker may be required to provide its intrinsic rigidity and can also be involved in structural switching via cis–trans proline isomerization. The proline at the P588 position is conserved in all known Kaiso proteins from vertebrates with sequenced genomes (Supplementary Figure S1). It is also conserved in the paralogous proteins ZBTB38 and ZBTB4 as well as in the ancestral Kaiso-like protein from the lamprey Petromyzon marinus, the most basal living vertebrate in which we were able to trace Kaiso orthologs (Figure 1d). Another proline, P577, is less conserved. It is absent in cartilaginous fishes (as determined by assessing the Kaiso sequence in the white shark Carcharodon carcharias), several birds (Supplementary Figure S1), and the human ZBTB38 protein (Figure 1c,d).

The P577 proline has an adjacent conserved serine, S578, which forms the H-bond with the DNA backbone in a complex with CpG-methylated oligonucleotides. Serine residues are common in polypeptides that contain prolines switched by prolyl-isomerases upon phosphorylation; however, such serines are typically located N-terminally to prolines [10]. It is possible that P577 in cis-conformation may prevent beta-sheet formation with the third zinc finger upon binding to DNA.

Finally, the Kaiso DNA-binding domain contains another proline (P523) that is located within the first beta-strand of the second zinc finger and is substituted for glutamine in the mouse. It is unlikely that this proline undergoes trans-to-cis conversion.

### 2.2. Molecular Dynamics Simulation Revealed the Importance of the Rigidity of the C-Terminal Extension Linker for the Overall Stability of the Kaiso–DNA Complex

**Figure 2:**
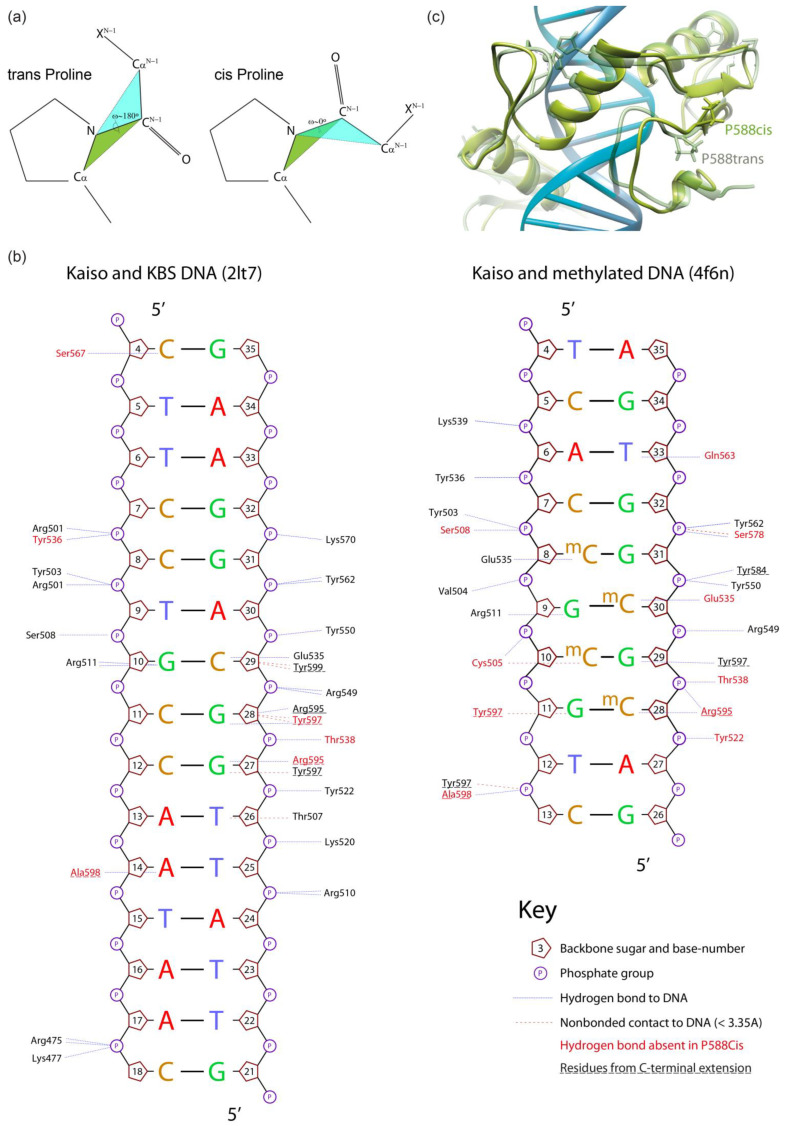
Comparison of 100 ns molecular dynamics simulation of wild-type and P588-altered Kaiso–DNA complexes (based on the PDB:2LT7 NMR structure of Kaiso with unmethylated DNA). (**a**) Schematic representation of cis- and trans-proline conformations. (**b**) Schematic representation of the effect of P588 trans to cis isomerization on the hydrogen bonds formed by Kaiso protein with Kaiso binding sequence or methylated DNA. Plots were drawn for initial structures using Nucplot [33]. (**c**) Overlay of wild-type and P588^cis^ Kaiso–DNA complexes.

**Figure 3:**
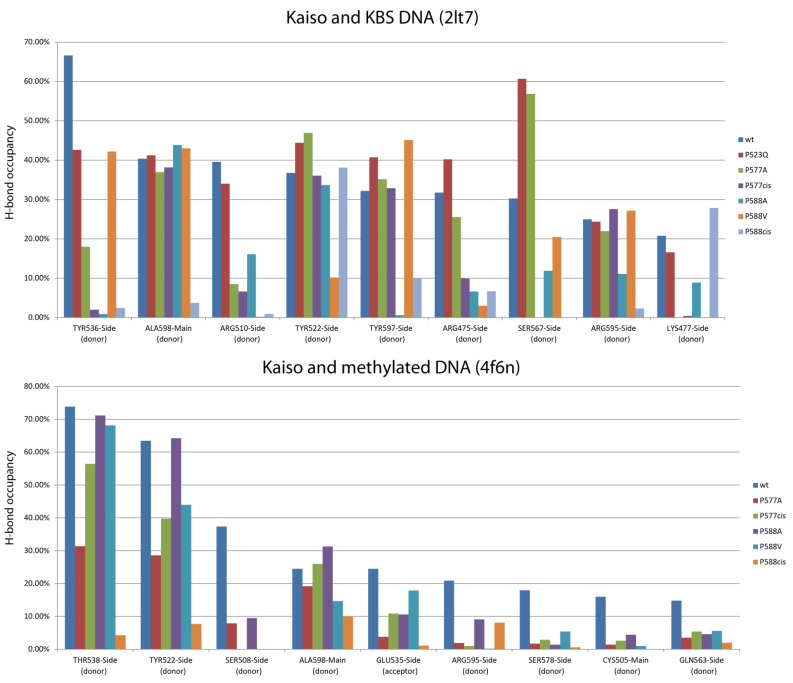
The plots of the presence of protein–DNA hydrogen bonds that weakened more than 2-fold (compared to wild-type protein) along the last 90 ns of 100 ns molecular dynamics trajectories of Kaiso complexes with KBS or CG-methylated DNA. Only bonds with over 15% presence in wild-type protein are shown. Complete data are shown in Supplementary Tables S2 (KBS) and S3 (methylated DNA). Several affected H-bonds (R595, Y597, and A598) make contact with the DNA minor groove and are formed by residues located in the C-terminal extension of the zinc-finger domain that is directly affected by the isomerization, while few bonds are formed by residues lying within the second zinc-finger domain (Y536 and T538), S567, which also forms hydrogen bonds, is in the third zinc-finger motif. Notably, most of these bonds are base-specific interactions; thus, their loss may result in a decrease in binding specificity (Figure 2b and Figure 3, Supplementary Table S2 and S3). However, most of the bonds located in the first and second zinc fingers involved in core sequence recognition [15] remain unchanged.

**Figure 4:**
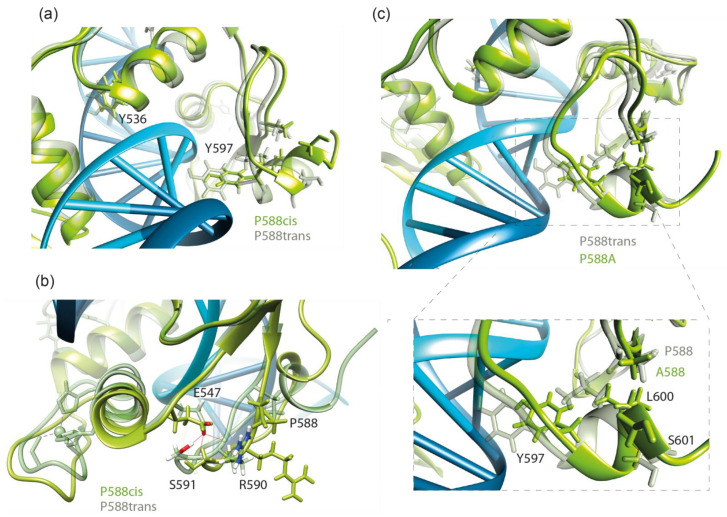
(**a**) The view of changes in the conformation of the Y536 and Y597 residues forming hydrogen bonds with DNA upon P588^cis^ isomerization at the end of a 100 ns molecular dynamics simulation. (**b**) The presence of the E547-R590/S591 hydrogen bond along molecular dynamics trajectories of wild-type and P588^cis^ Kaiso complexes with Kaiso binding sequence DNA. (**c**) The overlay of wild-type and P588A-substituted Kaiso–DNA complexes. The position of the P588-L600 hydrophobic contact is shown.

To analyze the possible impact of chain relaxation due to proline mutation to alanine or conversion to cis-conformation on Kaiso DNA binding, we performed MD simulations using known spatial structures of the Kaiso DNA-binding domain with methylated DNA (crystal structure, PDB: 4F6N) and a specific KBS (NMR structure, PDB: 2LT7). Spontaneous isomerization of an X-Pro peptide bond occurs slowly on a millisecond to second timescale [10,32]; thus, it cannot be observed in standard MD simulation. To simulate the proline interconversion, we applied a dihedral restraint to the ω angle between P588 and the preceding H587 to fix it at 0° corresponding to the cis conformation (Figure 2a). A similar restraint was independently applied to the ω angle between the P577 residue and the preceding D576 to simulate P577 cis-isomerization. Simulations were also performed for alanine substitutions to estimate the effect of chain relaxation; notably, these substitutions can be assayed in vitro. P523Q mutation was used as an additional control because it was not expected to impact H-bonds. Prior evaluation of longer MD simulation times of Kaiso P588A and P588^cis^ complexes with KBS DNA did not reveal significant differences from the first 100 ns of simulation (Supplementary Figure S2); thus, 100 ns trajectories were found to be sufficient and were used for all subsequent simulations. Analyses of RMSDs along the trajectories confirm the stability of molecular systems during simulation (Supplementary Figures S3 and S4). Results of protein–DNA H-bond presence analysis along the MD trajectories are shown in Figure 2b and Figure 3, while Supplementary Table S2 shows the complete results for the complex with KBS, and Supplementary Table S3 presents the results for methylated DNA. The P523Q substitution did not result in any unique changes, whereas the P588A substitution led to the loss of two H-bonds formed by Y536 and Y597 (Supplementary Table S2) in Kaiso–KBS complexes. The strongest effects were observed with the P588^cis^ simulation. Overall comparison of the wild-type and P588^cis^ Kaiso proteins with specific KBS DNA complexes at the end of a 100 ns simulation are shown in Figure 2c. P588 cis-isomerization led to further loss of Kaiso–KBS H-bonds in addition to the bonds formed by Y536 and Y597 that were predicted to be lost with the P588A substitution (Supplementary Tables S2 and S3, Figure 3 and Figure 4a). P577^cis^ led mostly to allosteric effects of losing a few H-bonds between zinc-finger residues and DNA without affecting residues within the extension, while its mutation to alanine had only subtle effects, with only one H-bond (mediated by K477) lost (Supplementary Table S2). In some cases, a few H-bonds increased their presence in mutant proteins; however, their presence did not exceed the 15% threshold which was used for wild-type proteins, and thus these alterations were not taken into account.

A significant alteration of the H-bonding network was also observed for the complex of Kaiso with methylated DNA. P588^cis^ altered bonds formed by many residues involved in methylcytosine recognition from both the zinc fingers and the extension (Supplementary Table S3, Figure 2b and Figure 3). P577^cis^ isomerization also led to the loss of some H-bonds and eventually resulted in the dissociation of the C-terminal extension from DNA at the end of a 100 ns simulation, which is reflected in the increasing RMSD (Supplementary Figure S4). When the simulation was continued for a further 400 ns, re-association was not observed. P577A had almost the same negative effects but did not lead to the dissociation of the C-terminal extension. Unexpectedly, we observed low occupancy of the H-bond between E535 and methylated bases, even with wild-type Kaiso (Supplementary Table S3). This interaction is considered one of the main determinants of methylated base binding specificity [15,34,35]; thus, low occupancy may suggest either the highly dynamic nature of this contact or possible inconsistencies in simulation. It should be noted that results obtained for Kaiso complexes with methylated DNA are not directly comparable to results from Kaiso–KBS complexes because different initial coordinates and different force fields were used for simulations.

According to Buck–Koehntop et al. [15], upon DNA binding, the C-terminal extension forms a structured loop stabilized through H-bonding interactions between the side chain of E547 and the backbone amides of R590/S591 as well as a series of nonpolar interactions with the helix of the second zinc finger (Figure 4b). We studied the presence of E547-R590/S591 H-bonds along MD trajectories for complexes of Kaiso with both KBS and methylated DNA (Table 1). Almost complete disappearance of this bond was observed only in the P588^cis^ simulation. Thus, the loss of H-bonds formed by residues within the second zinc finger (Y536 and T538) likely occurs due to an allosteric effect caused by the relaxation of the C-terminal extension located on the opposite side of the DNA helix. Local unfolding due to the relaxation of the polypeptide chain also results in the disappearance of the hydrophobic contact between P588 and L600, which likely leads to the loss of the Y597-DG28 base-specific H-bond (Figure 4c). The loss of this contact also occurs with the P588A mutation. Substitution of P588 with a residue with a large hydrophobic side chain would presumably restore the conformation; furthermore, it would restrain the movement of the polypeptide chain. Indeed, the MD simulation of the P588V substitution predicted the restoration of the contact with L600 and preservation of the H-bond between Y597 and KBS DNA (Figure 3, Supplementary Table S2). As a result, in silico P588V substitution rescued most of the negative effects of P588^cis^ and P588A on the interaction of the C-terminal extension with KBS DNA, although it affected several H-bonds formed by zinc fingers, mostly with the DNA sugar-phosphate backbone (Supplementary Table S2). In contrast to the interaction with KBS sites, for the complex of Kaiso with methylated DNA, the P588V substitution only partially rescued the negative impact of isomerization and had similar effects to P588A and P577^cis^ on the H-bonding network according to MD simulations (Figure 3, Supplementary Table S3).

Taken together, the simulation data revealed multiple allosteric effects of alterations in the C-terminal extension, suggesting that the DNA binding of Kaiso likely involves squeezing of DNA between the second zinc finger and the C-terminal extension of the third zinc finger; thus, loss of this extension or its rigidity due to proline mutation or isomerization affects the DNA-binding properties of the entire complex.

### 2.3. The Presence of the C-Terminal Extension and Restrained Mobility in Its Linker Are Required for Efficient Kaiso DNA Binding In Vitro

**Figure 5:**
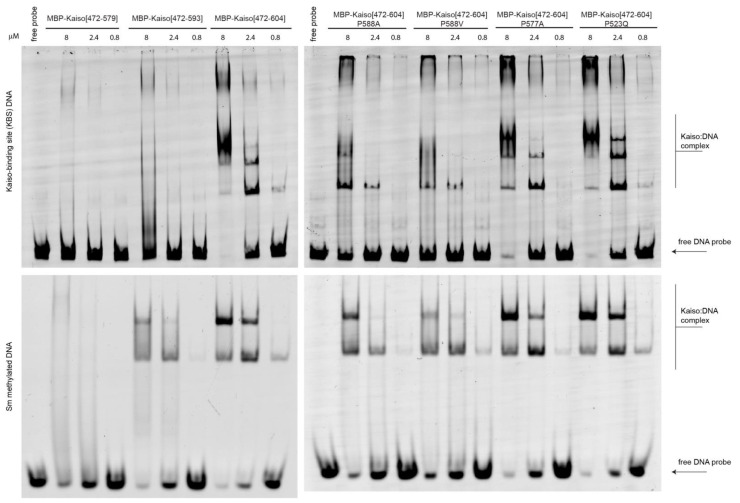
Electrophoretic mobility shift assay to assess the impact of P588 substitutions and deletions of the C-terminal extension on DNA-binding activity of Kaiso protein in vitro. Uncropped gels are shown in Supplementary Figure S6.

To confirm the results of MD simulations, we generated single amino acid substitutions along with deletion derivatives of the Kaiso DNA-binding domain and performed an electrophoretic mobility shift assay with both KBS and CG-methylated DNA oligonucleotides. Protein purity and quantity were further verified with protein electrophoresis, as shown in Supplementary Figure S5a. The absence of binding to unmethylated nonspecific sites was confirmed independently (Supplementary Figure S5b). The results shown in Figure 5 support the proposed mechanism of allosteric regulation of Kaiso activity by the C-terminal loop, which requires the presence of prolines in particular positions. In accordance with a previous report [14], deletion of the entire extension, including the first beta-strand (Δ579–604), completely abolished DNA binding. Deletion of the last part (Δ593–604), which forms few DNA contacts, also impaired DNA binding, especially to the KBS. The latter deletion retains the residues R590/S591 that form an H-bond with E547 of the second zinc finger, thus preserving intramolecular tension to some extent. Notably, residues 594–604 in the C-terminal extension are not absolutely required for DNA binding; rather, they stabilize interactions mediated by zinc fingers. The stronger effect of the Δ593–604 truncation on Kaiso binding to KBS sites compared with its binding to methylated oligos conflicts with the MD data suggesting a stronger impact on methyl-DNA binding. In contrast, P588A, which increases mobility in the extension linker, had a strong negative effect on KBS binding and a weak effect on methyl-DNA binding. It is possible that the presence of an altered C-terminal loop disturbs the complex to a greater extent than the complete absence of the loop, leading to the ambiguous effects observed. The P588V substitution also almost completely abolished Kaiso DNA-binding activity with KBS. Although this mutation restores the ability of the C-terminal extension to interact with specific DNA sites, it leads to a loss of chain rigidity and intramolecular tension required for efficient binding, which is reflected in the loss of several H-bonds formed by zinc fingers, as suggested by MD simulation (Supplementary Table S2). The P577A substitution did not decrease Kaiso binding affinity to either methylated or KBS DNA, in contrast to MD simulations that predicted that this substitution would influence binding of the Kaiso complex to methylated DNA. This discrepancy, along with the aforementioned low occupancy of the H-bond between E535 and methylated bases, may be attributed to some previously mentioned [36] inaccuracies in the CHARMM36m force field (which was used for MD simulations of Kaiso complexes with methylated DNA due to the built-in parameters for 5-methylcytosine) when applied to protein–DNA complexes.

As predicted, the P523Q substitution within the second zinc finger did not affect Kaiso binding affinity to either methylated or KBS DNA.

## 3. Discussion

Proline residues are important for protein conformational stability and can play a significant role in the regulation of the DNA-binding activity of transcription factors. In this study, we explored the significance of prolines within linkers connecting the C-terminal extension to zinc fingers of the Kaiso DNA-binding domain in DNA-binding activity and the possibility that DNA binding is regulated through proline trans to cis isomerization. The presence of the extension and its conformational rigidity provided by the presence of prolines were found to be critical both for efficient Kaiso DNA binding in vitro and for the preservation of the DNA–protein H-bonding network in MD simulations. According to MD simulations, P588 cis-isomerization results in the loss of not only the same H-bonds as alanine substitutions (which significantly decreased DNA-binding efficiency in vitro) but also a few additional H-bonds. Thus, it is likely that P588^cis^ would have a quite similar or stronger effect on the Kaiso–DNA interaction, resulting in a loss of DNA-binding efficiency. Implementing high forces to restrain the proline conformation could induce artificial structural disruptions; however, many of the affected H-bonds coincide between simulations of cis-proline and of its mutations to alanine or valine, suggesting that simulations of cis-proline are reliable. MD simulations using different force fields also led to quite similar results, further supporting the reliability of cis-proline modeling. Substitution of P588 with a large hydrophobic residue in silico could partially rescue the binding affinity with specific DNA sites, but was not sufficient for efficient DNA binding in vitro. Notably, such a substitution is not found in any vertebrate, emphasizing the requirement for proline at this position. This suggests that Kaiso DNA-binding activity may be regulated by a cis–trans proline switch mediated by proline-isomerase family proteins. Since MD simulations were carried out with existing Kaiso–DNA complexes, MD simulation of P588 in cis-conformation revealed vast structural rearrangements that weakened even existing DNA interactions. Isomerization of prolines to cis-conformation by prolyl-isomerase in solution may inactivate the ability of the C-terminal extension to acquire an ordered conformation through interaction with the second zinc finger and prevent stabilization of the Kaiso–DNA complex, resulting in the complete inability of Kaiso to bind its sites. A common phosphorylation-mediated mechanism for zinc finger eviction from DNA during mitosis has been described [37], and the action of prolyl-isomerases can contribute to this mechanism. Another proline in the C-terminal extension, P577, seems to have a weak impact on DNA binding; nonetheless, it may contribute to efficient switching of the extension conformation in solution and can likely be regulated through phosphorylation of the adjacent serine. Notably, however, this serine is located C-terminally to the proline, which is not typical for the currently described phosphorylation-dependent switching mechanism [10]. P577 is substituted either to threonine in the white shark *C. carcharias* and the bird *Junco hyemalis* or to histidine in the human Kaiso paralog ZBTB38. Both of these residues allow much greater conformational flexibility than proline, which may lead to a higher dissociation rate of the protein–DNA complex. In the Kaiso protein from *C. carcharias*, this substitution is accompanied by mutations of adjacent residues to positively charged residues, which may compensate for the altered H-bonding pattern.

MD simulation revealed multiple allosteric effects of the alterations in the C-terminal extension on the DNA-binding properties of zinc fingers. These findings suggest the possible requirement of intramolecular tension for efficient DNA binding and prevention of complex dissociation. Such a mechanism may be widely exploited by C2H2 proteins, given that prolines are often present within linkers between individual C2H2 domains. Notably, both inter-C2H2 linkers of Kaiso lack prolines; thus, a single cis–trans proline switch could regulate the activity of the entire domain. The existence of similar extensions may be widespread among zinc finger proteins; however, they are difficult to predict because they would likely remain unstructured without DNA. Further in vivo studies are required to elucidate the role of the C-terminal loop and possible proline switching in the cellular functions of Kaiso.

## 4. Materials and Methods

### 4.1. Plasmids and Cloning

cDNAs of Kaiso derivatives were PCR-amplified using corresponding primers (see Supplementary Table S1) and cloned into a modified pMAL-C5x vector (New England Biolabs, Ipswich, MA, USA) in frame with MBP-tag. PCR-directed mutagenesis was used to create constructs expressing mutant proteins using mutagenic primers (see Supplementary Table S1).

### 4.2. Protein Expression and Purification

All chemical reagents are Applichem (Darmstadt, Germany) unless otherwise mentioned. Purification of MBP-tagged proteins was performed with Immobilized Amylose Agarose (New England Biolabs, Ipswich, MA, USA) in buffer A (20 mM Tris (pH 7.5); 150 mM NaCl; 10mM MgCl_2_; 0.1 mM ZnCl_2_; 0.1% NP40; 10% [*w*/*w*] glycerol; 1 mM DTT). BL21 cells transformed with plasmids expressing MBP-fused proteins were grown in LB media to an A600 of 1.0 at 37 °C and then induced with 1 mM IPTG at 18 °C overnight. ZnCl_2_ was added to a final concentration of 100 μM before induction. Cells were disrupted by sonication in 1 mL of buffer A, and after centrifugation, lysate was applied to pre-equilibrated resin for 10 min at +4 °C; subsequently, the resin was washed four times with 1 mL of buffer A containing 500 mM NaCl and bound proteins were eluted with 40 mM maltose, 20 mM Tris (pH 8.0), 200 mM NaCl, and 1mM DTT for 15 min. 

### 4.3. Electrophoretic Mobility Shift Assay

Samples (20 μL) containing 25 nM duplex FAM-labeled methylated DNA or Cy5-labeled KBS DNA (Evrogen, Moscow, Russia) were incubated with MBP (maltose-binding protein)-tagged Kaiso derivatives for 30 min at room temperature in buffer A with added 120 ng/μL Poly(dI-dC). Electrophoresis was performed in 0.5× Tris·borate buffer at 250 V for 30 min at 4 °C on 5% [*m*/*w*] nondenaturing acrylamide gels. Gels were scanned on the ChemiDoc MP Imaging system (Bio-Rad, Hercules, CA, USA).

### 4.4. Molecular Dynamics Simulations

All MD simulations were performed using the molecular simulation package GROMACS 2021.5 (Groningen, The Netherlands) [38] at a CUDA-accelerated workstation using the AMBER force fields ff19SB [39] for protein and BSC1 [40] for DNA for the Kaiso complex with KBS DNA and the CHARMM36m force field [41] for the Kaiso complex with methylated DNA at the same experimental temperature (298.15 K) and pressure (1 atm). The initial conformations for the simulations come from the NMR structure of Kaiso zinc fingers and an unmethylated Kaiso DNA-binding site (PDB 2LT7) and the crystal structure of Kaiso zinc fingers with methylated DNA (PDB 4F6N). These systems were solvated in a cube of TIP3P water molecules and 150 mM Na^+^ and Cl^-^ ions with the shortest distance of solute to box surfaces of 1.5 nm. Minimization simulation was performed using the steepest-descent energy minimization method. All production simulations were performed under periodic boundary conditions in the constant volume, temperature (310 K), pressure (1 atm), and number of particles (72,563 atoms for wild-type Kaiso complex with KBS DNA; 70,116 atoms for wild-type complex of methylated DNA) ensembles. Before the production runs, sequential 125 ps equilibration processes in the ensembles with constant volume, temperature (Nose–Hoover thermostat), and number of particles (NVT); then with constant volume, temperature, number of particles, and pressure (Parrinello–Rahman barostat), (NPT) were performed to adjust the systems for the desired temperatures and volumes. In all simulations, the Linear Constraint Solvent (LINCS) algorithm was used to constrain bonds involving hydrogen. The nonbonded cutoff for evaluating electrostatic and van der Waals forces was set to 1.0 nm. To deal with long-range electrostatic interactions, the Particle mesh Ewald (PME) algorithm was used with the default settings, including a real space grid of 0.12 nm. The coordinates were written to file every 100 ps. The CHARMM-GUI interface [42] was used to set up initial configuration files and to introduce point mutations. To simulate cis-proline conformation, after the energy minimization, the dihedral restraint was implied: a constant force of 500 kJ/mol/rad^2^ was applied to restrain the ω angle between the proline and the preceding residue to 0°. H-bond and RMSD analysis were performed with VMD software (Urbana, IL, USA) [43]. To calculate H-bonds presence, 900 frames starting from 10 ns of production simulation were used; the donor-acceptor distance cutoff was 3.0 Å and the angle cutoff was +/− 20 degrees from linearity.

## 5. Conclusions

Both MD simulations and in vitro binding assays indicate that the presence of prolines in particular positions in trans conformation within the C-terminal extension is essential for efficient Kaiso DNA binding. MD data suggest that cis–trans proline switching may be involved in the regulation of Kaiso binding to its sites. Alterations within the C-terminal extension result in multiple allosteric effects, which can be explained mechanistically by the presence of intramolecular tension squeezing the DNA chain. Further studies are required to reveal the role of possible proline switching in vivo and to explore the existence of similar extensions among zinc finger proteins.

## Figures and Tables

**Table 1:** The presence of the E547-R590/S591 hydrogen bond along last 90 ns of 100 ns molecular dynamics trajectories of Kaiso complexes with Kaiso binding sequence and methylated DNA. Grey fill indicates H-bonds weakened 5× times or more compared to wild-type protein.

Donor	Acceptor	wt	P523Q	P577^cis^	P588A	P588V	P588^cis^
KBS DNA							
SER591-Main	GLU547-Side	33.87%	39.76%	35.46%	32.77%	33.71%	1.60%
SER591-Side	GLU547-Side	77.52%	60.34%	63.94%	48.15%	70.32%	26.65%
ARG590-Main	GLU547-Side	29.67%	50.65%	37.36%	43.16%	39.25%	0.10%
methylated DNA							
SER591-Main	GLU547-Side	46.35%	ND	27.07%	48.55%	45.95%	0.10%
SER591-Side	GLU547-Side	80.72%	ND	70.13%	83.22%	79.12%	10.29%
ARG590-Main	GLU547-Side	36.06%	ND	32.67%	31.07%	35.86%	2.30%

## Data Availability

Not applicable.

## Supplementary Materials

The supporting information can be downloaded at: https://www.mdpi.com/article/10.3390/ijms232415494/s1.
