# Supplementary material for: The Essential Role of Prolines and Their Conformation in Allosteric Regulation of Kaiso Zinc Finger DNA-Binding Activity by the Adjacent C-Terminal Loop
Source: Int J Mol Sci. 2022 Dec 7;23(24):15494. doi: 10.3390/ijms232415494 (PMC9779254; doi:10.3390/ijms232415494)

Supplementary information for:

The Essential Role of Prolines and Their Conformation in Allosteric Regulation of Kaiso Zinc Finger DNA-Binding Activity by the Adjacent C-Terminal Loop

Elena Belova [1], Oksana Maksimenko [2], Pavel Georgiev [1,*] and Artem Bonchuk [1,2,*]

[1] Department of the Control of Genetic Processes, Institute of Gene Biology Russian Academy of Sciences, 34/5 Vavilov St., Moscow 119334, Russia
[2] Center for Precision Genome Editing and Genetic Technologies for Biomedicine, Institute of Gene Biology, Russian Academy of Sciences, 34/5 Vavilov St., Moscow 119334, Russia
* Correspondence: georgiev_p@mail.ru (P.G.); bonchuk_a@genebiology.ru (A.B.)

Supplementary Table S1. Oligonucleotides used for cloning and EMSA. Protein binding sites are shown in bold.

1.1. Oligonucleotides used for cloning

Kaiso_P523Q_d	GTATCAGTGCCGTTACTGT
Kaiso_P523Q_r	ACAGTAACGGCACTGATAC
Kaiso_P577A_d	GATGCTTCTGGGGACTCA
Kaiso_P577A_r	TGAGTCCCCAGAAGCATC
Kaiso_P588A_d	CATGCATGCAGGTCTTTAC
Kaiso_P588A_r	GTAAAGACCTGCATGCATG
Kaiso_P588V_d	CATGTATGCAGGTCTTTAC
Kaiso_P588V_r	GTAAAGACCTGCATACATG
Kaiso_579_r	TTGTCGACTTACCCAGAAGGATCTTGACTA
Kaiso_593_r	TTGTCGACTTATTGTAAAGACCTGCATGGAT
Kaiso_604_r	TTGTCGACTTATGATCTATCGGAAAGATATGC
Kaiso_472_d	TTGAATTCGCAAACAAACGTATGAAAGTA

1.2. DNA probes for EMSA

Kaiso BSx3

AACAGCTATGACCATGATTACGCCAAGCTCGAAATTAACCCTCACTAAAGGGAACAAAAGCTGGAGCTCCACCG
CGGTGGCGGCCGCTCTAGAACTAGTGCT**TCCTGCCA**ATAACGGTGCT**TCCTGCCA**ATAACGGTGCT**TCCTGCCA**
ATAAGCTTATCGATACCGTCGACCTCGAGGGGGGGCCCGGTACCCAATTCGCCCTATAGTGAGTCGTATTACAA
TTCACTGGCCGTCGTTTTAC

Pita BSx5 as nonspecific DNA

GTAAAACGACGGCCAGTGAGCGCGCGTAATACGACTCACTATAGGGCGAATTGGGTACCGGGCCCCCCCTCGA
GGTCGACGGTATCGATAAGCTTGATCTTT**AGCCAAGACGCGAACCCGAATCCG**AAACTTT**AGCCAAGACGCGA**
ACCCGAATCCGAAACTTT**AGCCAAGACGCGAACCCGAATCCG**AAACTT**AGCCAAGACGCGAACCCGAATCCG**
AAACTTT**AGCCAAGACGCGAACCCGAATCCG**AAGATCTAATATCGAATTCCTGCAGCCCGGGGGATCCACTAG
TTCTAGAGCGGCCGCCACCGCGGTGGAGCTCCAGCTTTTGTTCCCTTTAGTGAGGGTTAATTTCGAGCTTGGCG
TAATCATGGTCATAGCTGTT

Supplementary Table S2. The presence of protein–DNA hydrogen bonds along last 90 ns of 100 ns molecular dynamics trajectories of Kaiso complexes with Kaiso binding sequence DNA. Only bonds with over 20% presence in wild-type protein are shown. Grey fill indicates H-bonds weakened 5x times or more compared to wild-type protein. Light grey fill indicates H-bonds weakened 2-5x times compared to wild-type protein.

donor	acceptor	wt	P523Q	P577A	P577^{cis}	P588A	P588V	P588^{cis}
TYR550-Side	DA30-PO4	85.41%	81.04%	81.72%	85.11%	85.91%	88.22%	68.06%
TYR562-Side	DG31-PO4	77.72%	74.75%	76.12%	67.03%	73.83%	74.15%	77.54%
THR538-Side	DG28-PO4	72.43%	72.46%	69.73%	61.04%	55.14%	53.09%	16.27%
TYR584-Side	DA30-PO4	71.73%	62.67%	68.73%	73.73%	68.73%	72.65%	66.77%
ARG549-Side	DC29-PO4	67.93%	85.83%	67.03%	60.24%	87.81%	68.06%	83.43%
TYR536-Side	DC8-PO4	66.63%	42.61%	17.98%	2.00%	0.90%	42.22%	2.40%
SER508-Side	DG10-PO4	63.84%	57.78%	62.14%	61.94%	66.33%	71.46%	41.82%
TYR503-Side	DT9-PO4	54.65%	69.16%	63.54%	66.33%	69.63%	65.17%	66.97%
ARG511-Side	DG10-Base	45.65%	60.98%	61.14%	60.84%	62.54%	49.50%	45.51%
ALA598-Main	DA14-Base	40.36%	41.22%	36.96%	38.16%	43.86%	43.01%	3.69%
ARG510-Side	DT25-PO4	39.56%	34.03%	8.49%	6.59%	16.08%	0.20%	0.90%
TYR522-Side	DG27-PO4	36.76%	44.41%	46.95%	36.06%	33.67%	10.18%	38.12%
TYR597-Side	DG28-Base	32.17%	40.72%	35.16%	32.87%	0.60%	45.11%	10.08%
ARG475-Side	DC18-PO4	31.77%	40.22%	25.57%	9.89%	6.59%	2.99%	6.69%
SER567-Side	DC4-Base	30.27%	60.68%	56.84%	0.00%	11.89%	20.46%	0.00%
ARG501-Side	DT9-PO4	25.37%	34.03%	20.58%	22.88%	25.37%	24.35%	24.45%
ARG595-Side	DG27-Base	24.98%	24.35%	21.98%	27.57%	11.09%	27.15%	2.30%
DC29-Base	GLU535-Side	22.08%	39.92%	39.66%	42.86%	42.66%	19.16%	44.71%
LYS477-Side	DC18-PO4	20.78%	16.57%	0.10%	0.40%	8.89%	0.10%	27.84%

Supplementary Table S3. The presence of protein–DNA hydrogen bonds along last 90 ns of 100 ns molecular dynamics trajectories of Kaiso complexes with CG-methylated DNA. Only bonds with over 15% presence in wild-type protein (and the bond E535-5MC8, which was previously found to be important for specific methyl-DNA recognition) are shown. Designations are the same as in Supplementary Table 2.

donor	acceptor	wt	P577A	P577^{cis}	P588A	P588V	P588^{cis}
ARG549-Side	5MC30-PO4	100.00%	100.00 %	92.71%	100.00 %	100.00%	100.00 %
ARG511-Side	DG9-Base	100.00%	56.74%	43.66%	90.11%	70.83%	33.57%
TYR503-Side	5MC8- PO4	83.62%	79.82%	65.13%	80.02%	70.73%	82.52%
THR538-Side	DG29-PO4	73.83%	31.37%	56.44%	71.13%	68.13%	4.30%
TYR584-Side	DG31-PO4	73.13%	79.82%	63.74%	77.72%	76.72%	76.52%
TYR562-Side	DG32- PO4	71.23%	70.93%	58.14%	64.94%	69.53%	65.73%
TYR550-Side	DG31-Side	70.63%	68.93%	65.43%	69.03%	69.03%	60.04%
TYR522-Side	5MC28- PO4	63.44%	28.57%	39.76%	64.24%	43.96%	7.69%
SER508-Side	5MC8- PO4	37.36%	7.89%	0.0%	9.49%	0.10%	0.0%
VAL504-Main	DG9- PO4	28.57%	52.05%	45.65%	47.35%	51.15%	61.64%
ALA598-Main	DC13- PO4	24.48%	19.18%	25.97%	31.27%	14.69%	10.09%
5MC30-Side	GLU535-Side	24.48%	3.80%	10.89%	10.59%	17.88%	1.10%
ARG595-Side	DG29-PO4	20.88%	1.90%	1.00%	9.09%	0.20%	8.09%
ARG595-Side	5MC28-Base	19.68%	28.17%	29.57%	26.57%	28.77%	17.58%
SER578-Side	DG32- PO4	17.98%	1.70%	2.90%	1.40%	5.39%	0.60%
TYR597-Side	DG29-Base	16.48%	16.58%	15.88%	26.17%	18.18%	25.47%
CYS505-Main	DG9-PO4	15.98%	1.40%	2.60%	4.40%	1.00%	0.0%
GLN563-Side	DT33-Base	14.79%	3.50%	5.39%	4.60%	5.59%	2.00%
…							
5MC8-Side	GLU535-Side	1.70%	6.09%	6.19%	9.99%	1.50%	1.20%

Supplementary Figure S1. Multiple sequence alignment of C-terminal extensions of Kaiso proteins from 400 species of Vertebrates.

	P577 P588
Accipiter_nisus	HSQDPSGDTKLYRLHPCRSLQ
Acinonyx_jubatus	HSQDPSGDAKLYRLHPCRSLQ
Acrocephalus_arundinaceus	HSQDPSGDTKLYRLHPCRSLQ
Aegotheles_bennettii	HSQDPSGDNKLYRLHPCRSLQ
Ailuropoda_melanoleuca	HSQDPSGDSKLYRLHPCRSLQ
Alca_torda	HSQDPSGDTKLYRLHPCRSLQ
Aleadryas_rufinucha	HSQDPSGDTKLYRLHPCRSLQ
Alligator_mississippiensis	HSQDPSGDAKLYRLHPCRSLQ
Alligator_sinensis	HSQDPSGDAKLYRLHPCRSLQ
Alopecoenas_beccarii	HSQDPSGDTKLYRLNPCRSLQ
Anas_platyrhynchos	HSQDPSGDTKLYRLHPCRSLQ
Anas_platyrhynchos_platyrhynchos	HSQDPSGDTKLYRLHPCRSLQ
Anas_zonorhyncha	HSQDPSGDTKLYRLHPCRSLQ
Anhinga_anhinga	HSQDPSGDTKLYRLHPRRSLR
Anolis_carolinensis	HSQDPSGESKHYRLHPCRSLQ
Anser_brachyrhynchus	HSQDPSGDTKLYRLHPCRSLQ
Anser_cygnoid	HSQDPSGDTKLYRLHPCRSLQ
Anthoscopus_minutus	HSQDPSGDTKLYRLHPCRSLQ
AOrycteropus_afer_afer	HSQDPSGDAKLYRLHPCRSLQ
Apaloderma_vittatum	HSQDPSGDTKLYRLHPCRSLQ
Aphelocoma_coerulescens	HSQDPSGDTKLYRLHPCRSLQ
Aptenodytes_forsteri	HSQDPSGDTKLYRLHPCRSLQ
Aptenodytes_patagonicus	HSQDPSGDTKLYRLHPCRSLQ
Aquila_chrysaetos_chrysaetos	HSQDPSGDTKLYRLHPCRSLQ
Aramus_guarauna	HSQDPSGDTKLYRLHPCRSLQ
Ardeotis_kori	HSQDPSGDTKLYRLHPCRSLQ
Arenaria_interpres	HSQDPSGDTKLYRLHPCRSLQ
Asarcornis_scutulata	HSQDPSGDTKLYRLHPCRSLQ
Athene_cunicularia	HSQDPSGDTKLYRLHPCRSLQ
Atlantisia_rogersi	HSQDPSGDTKLYRLHPCRSLQ
Aythya_fuligula	HSQDPSGDTKLYRLHPCRSLQ
Balaeniceps_rex	HSQDPSGDTKLYRLHPCRSLQ
Balaenoptera_acutorosUrsus_maritimus	HSQDPSGDSKLYRLHPCRSLQ
Balaenoptera_musculus	HSQDPSGDSKLYRLHPCRSLQ
Balaenoptera_physalus	HSQDPSGDSKLYRLHPCRSLQ
Balearica_regulorum_gibbericeps	HSQDPSGDTKLYRLHPCRSLQ
Baryphthengus_martii	HSQDPSGDTKLYRLHPCRSLQ
Bison_bison_bison	HSQDPSGDSKLYRLHPCRSLQ
Bombycilla_garrulus	HSQDPSGDTKLYRLHPCRSLR
Bos_indicus_x_Bos_taurus	HSQDPSGDSKLYRLHPCRSLQ
Bos_mutus	HSQDPSGDSKLYRLHPCRSLQ
Bos_taurus	HSQDPSGDSKLYRLHPCRSLQ
Bubo_bubo	HSQDPSGDTKLYRLHPCRSLQ
Buphagus_erythrorhynchus	HSQDPSGDTKLYRLHPCRSLR
Burhinus_bisSinosuthora_webbiana	HSQDPSGDTKLYLLHPCRSLQ
Buteo_japonicus	HSQDPSGDTKLYRLHPCRSLQ
Cairina_moschata_domestica	HSQDPSGDTKLYRLHPCRSLQ
Calcarius_ornatus	HSQDPSGDTKLYRLHPCRSLQ
Calidris_pygmaea	HSQDPSGDTKLYRLHPCRSLQ
Callaeas_wilsoni	HSQDPSGDTKLYRLHPCRSLQ
Callorhinus_ursinus	HSQDPSGDSKLYRLHPCRSLQ
Caloenas_nicobarica	HSQDPSGDTKLYRLNPCRSLQ
CalonecFregetta_grallaria	HSQDPSGDTKLYRLHPCRSLQ
Camelus_dromedarius	HSQDPSGDSKLYRLHPCRSLQ
Camelus_ferus	HSQDPSGDSKLYRLHPCRSLQ
Campylorhamphus_procurvoides	HSQDPSGDTKLYRLHPCRSLQ
Canis_lupus_dingo	HSQDPSGDSKLYRLHPCRSLQ

Canis_lupus_familiaris	HSQDPSGDSKLYRLHPCRSLQ
Canis_lupus_familiaris_0	HSQDPSGDSKLYRLHPCRSLQ
Canis_lupus_familiaris_1	HSQDPSGDSKLYRLHPCRSLQ
Capra_hircus	HSQDPSGDSKLYRLHPCRSLQ
Cardinalis_cardinalis	HSQDPSGDTKLYRLHPCRSLQ
Cariama_cristata	HSQDPSGDTKLYRLHPCRSLQ
Catagonus_wagneri	HSQDPSGDSKLYRLHPCRSLQ
Catharus_fuscescens	HSQDPSGDTKLYRLHPCRSLQ
Catharus_ustulatus	HSQDPSGDTKLYRLHPCRSLQ
Cavia_porcellus_	HSQDPSGDSKLYRLHPCKSLQ
Cephalopterus_ornatus	HSQDPSGETKLYRLHPCRSLQ
Cepphus_grylle	HSQDPSGDTKLYRLHPCRSLQ
Certhia_brachydactyla	HSQDPSGDTKLYRLHPCRSLQ
Certhia_familiaris	HSQDPSGDTKLYRLHPCRSLQ
Cettia_cetti	HSQDPSGDTKLYRLHPCRSLQ
Chaetorhynchus_papuensis	HSQDPSGDTKLYRLHPCRSLQ
Charadrius_vociferus	HSQDPSGDTKLYRLHPCRSLQ
Chauna_torquata	HSQDPSGDAKLYRLHPCRSLQ
Chelonia_mydas	HSQDPSGDTKLYRLHPCRSLQ
Chelonoidis_abingdonii	HSQDPSGDTKLYRLHPCRSLQ
Chelydra_serpentina	HSQDPSGDTKLYRLHPCRSLQ
Chinchilla_lanigera	HSQDPSGDSKLYRLHPCKSLQ
Chlamydotis_macqueenii	HSQDPSGDTKLYRLHPCRSLQ
Chloebia_gouldiae	HSQDPSGDTKLYRLHPCRSLQ
Chloropsis_cyanopogon	HSQDPSGNTKLYRLHPCRSLQ
Chloropsis_hardwickii	HSQDPSGNTKLYRLHPCRSLQ
Chordeiles_acutipennis	HSQDPSGDTKLYRLHPCRSLQ
Chroicocephalus_maculipennis	HSQDPSGDTKLYRLHPCRSLQ
Chrysemys_picta_bellii	HSQDPSGDTKLYRLHPCRSLQ
Chunga_burmeisteri	HSQDPSGDTKLYRLHPCRSLQ
Ciccaba_nigrolineata	HSQDPSGDTKLYRLHPCRSLQ
Ciconia_maguari	HSQDPSGDTKLYRLHPCRSLQ
Cinclus_mexicanus	HSQDPSGNTKLYRLHPCRSLQ
Circaetus_pectoralis	HSQDPSGDTKLYRLHPCRSLQ
Cisticola_juncidis	HSQDPSGDTKLYRLHPCRSLQ
Climacteris_rufus	HSQDPSGDTKLYRLHPCRSLQ
Cnemophilus_loriae	HSQDPSGDTKLYRLHPCRSLQ
Cochlearius_cochlearius	HSQDPSGDTKLYRLHPCRSLQ
Columbina_picui	HSQDPSGDTKLYRLNPCRSLH
Copsychus_sechellarum	HSQDPSGDTKLYRLHPCRSLQ
Corvus_brachyrhynchos	HSQDPSGDTKLYRLHPCRSLQ
Corvus_moneduloides	HSQDPSGDTKLYRLHPCRSLQ
Corythaeola_cristata	HSQDPSGDTKLYRLHPCRSLQ
Corythaixoides_concolor	HSQDPSGETKLYRLHPCRSLQ
Crocodylus_porosus	HSQDPSGDAKLYRLHPCRSLQ
Crocuta_crocuta	HSQDPSGDAKLYRLHPCRSLQ
Cyanistes_caeruleus	HSQDPSGDTKLYRLHPCRSLQ
Cyanoderma_ruficeps	HSQDPSGDTKLYRLHPCRSLQ
Cyanoderma_ruficeps_0	HSQDPSGDTKLYRLHPCRSLQ
Daphoenositta_chrysoptera	HSQDPSGDTKLYRLHPCRSLQ
Dasyornis_broadbenti	HSQDPSGDTKLYRLHPCRSLQ
Dicaeum_eximium	HSQDPSGDTKLYRLHPCRSLQ
Dicrurus_megarhynchus	HSQDPSGDTKLYRLHPCRSLQ
Donacobius_aVidua_chalybeata	HSQDPSGDTKLYRLHPCRSLQ
Dromas_ardeola	HSQDPSGDTKLYRLHPCKSLQ
Drymodes_brunneopygia	HSQDPSGDTKLYRLHPCRSLQ
Dryoscopus_gambensis	HSQDPSGDTKLYRLHPCRSLQ
Edolisoma_coerulescens	HSQDPSGDTKLYRLHPCRSLQ
Egretta_garzetta	HSQDPSGDTKLYRLHPCRSLQ
Elachura_formosa	HSQDPSGDTKLYRLHPCRSLQ
Emberiza_fucata	HSQDPSGDTKLYRLHPCRSLQ
Enhydra_luDelphinapterus_leucas	HSQDPSGDSKLYRLHPCRSLQ
Equus_asinus_asinus	HSQDPSGDSKLYRLHPCRSLQ
Erithacus_rubecula	HSQEPSGDTKLYRLHPCRSLQ

Erpornis_zantholeuca	HSQDPSGDTKLYRLHPCRSLQ
Erythrocercus_mccallii	HSQDPSGDTKLYRLHPCRSLQ
Eschrichtius_robustus	HSQDPSGDSKLYRLHPCRSLQ
Eudyptes_chrysocome	HSQDPSGDTKLYRLHPCRSLQ
Eudyptes_filholi	HSQDPSGDTKLYRLHPCRSLQ
Eudyptes_robustus	HSQDPSGDTKLYRLHPCRSLQ
Eudyptes_sclateri	HSQDPSGDTKLYRLHPCRSLQ
Eudyptula_minor	HSQDPSGDTKLYRLHPCRSLQ
Eulacestoma_nigropectus	HSQDPSGDTKLYRLHPCRSLQ
Eurypyga_helias	HSQDPSGDTKLYRLLPCRSLQ
Eurystomus_gularis	HSQDPSGDTKLYRLHPCRSLQ
Falco_tinnunculus	HSQDPSGDTKLYRLHPCRSLQ
Falcunculus_frontatus	HSQDPSGDTKLYRLHPCRSLQ
Felis_catus	HSQDPSGDSKLYRLHPCRSLQ
Ficedula_albicollis	HSQDPSGDTKLYRLHPCRSLQ
Formicarius_rufipectus	HSQDPSGDTKLYRLHPCRSLR
Fregata_magnificens	HSQDPSGDTKLYRLHPCRSLQ
Fukomys_damarensis	HSQDPSGDSKLYRLHPCKSLQ
Fulmarus_glacialis	HSQDPSGDTKLYRLHPCRSLQ
Furnarius_figulus	HSQDPSGDTKLYRLHPCRSLQ
Gavia_stellata	HSQDPSGDTKLYRLHPCRSLQ
GeoGeoMyotis_myotis	HSQDPSGDSKLYRLHPCRSLQ
Geospiza_fortis	HSQDPSGDTKLYRLHPCRSLQ
Geospiza_parvula	HSQDPSGDTKLYRLHPCRSLQ
Glareola_pratincola	HSQDPSGDTKLYRLHPCKSLQ
Glaucidium_brasilianum	HSQDPSGDTKLYRLHPCRSLQ
Gopherus_agassizii	HSQDPSGDTKLYRLHPCRSLQ
Gopherus_evgoodei	HSQDPSGDTKLYRLHPCRSLQ
Gorilla_gorilla_gorilla	HSQDPSGDSKLYRLHPCKSLQ
Grallaria_varia	HSQDPSGDTKLYRLHPCRSLQ
Grantiella_picta	HSQDPSGDTKLYRLHPCRSLR
Grus_americana	HSQDPSGDTKLYRLHPCRSLQ
Gulo_gulo	HSQDPSGDSKLYRLHPCRSLQ
Gymnorhina_tibicen	HSQDPSGDTKLYRLHPCRSLQ
Halcyon_senegalensis	HSQDPSGDTKLYRLHPCRSLQ
Haliaeetus_albicilla	HSQDPSGDTKLYRLHPCRSLQ
Heliornis_fulica	HSQDPSGDTKLYRLHPCRSLQ
Hemiprocne_comata	HSQDPSGDTKLYRLHPCRSLQ
Herpetotheres_cachinnans	HSQDPSGDTKLYRLHPCRSLQ
Himantopus_himantopus	HSQDPSGDTKLYRLHPCRSLQ
Hippolais_icterina	HSQDPSGDTKLYRLHPCRSLQ
Hipposideros_armiger	HSQDPSGESKLYRLHPCRSLQ
Hirundo_rustica_rustica	HSQDPSGDTKLYRLHPCRSLQ
Homo_sapiens	HSQDPSGDSKLYRLHPCKSLQ
Horornis_vulcanius	HSQDPSGDTKLYRLHPCRSLQ
Hypotaenidia_okinawae	HSQDPSGDTKLYRLHPCRSLQ
Ibidorhyncha_sThryothorus_ludovicianus	HSQDPSGETKMYRLHPCRSLQ
Ifrita_kowaldi	HSQDPSGDTKLYRLHPCRSLQ
Irena_cyanogasAnseranas_semipalmata	HSQDPSGDTKLYRLHPCRSLQ
Jacana_jacana	HSQDPSGDTKLYRLHPCRSLQ
Junco_hyemalis	HSQDTSGDTKLYRLHPCRSLQ
Lamprotornis_superbus	HSQDPSGDTKLYRLHPCRSLR
Lanius_ludovicianus	HSQDPSGDTKLYRLHPCRSLQ
Larus_smithsonianus	HSQDPSGDTKLYRLHPCRSLQ
Leiothrix_lutea	HSQDPSGDTKLYRLHPCRSLQ
Lepidothrix_coronata	HSQDPSGDTKLYRLHPCRSLQ
Leptocoma_aspasia	HSQDPSGDTKLYRLHPCRSLQ
Leptonychotes_weddellii	HSQDPSGDSKLYRLHPCRSLQ
Leptosomus_discolor	HSQDPSGDTKLYRLHPCRSLQ
Leucopsar_rothschildi	HSQDPSGDTKLYRLHPCRSLR
Limosa_lapponica_baueri	HSQDPSGDTKLYRLHPCRSLQ
Lipotes_vexillifer	HSQDPSGDSKLYRLHPCRSLQ
Locustella_ochotensis	HSQDPSGDTKLYRLHPCRSLQ
Lonchura_striata_domestica	HSQDPSGDTKLYRLHPCRSLQ

Lophotis_ruficrista	HSQDPSGDTKLYRLHPCRSLQ
Loxia_curvirosAgelaius_phoeniceus	HSQDPSGDTKLYRLHPCRSLQ
Loxia_leucoptera	HSQDPSGDTKLYRLHPCRSLQ
Lynx_canadensis	HSQDPSGDAKLYRLHPCRSLQ
Lynx_pardinus	HSQDPSGDAKLYRLHPCRSLQ
Machaerirhynchus_nigripectus	HSQDPSGDTKLYRLHPCRSLQ
Malurus_cyaneus_samueli	HSQDPSGDTKLYRLHPCRSLQ
Malurus_elegans	HSQDPSGDTKLYRLHPCRSLQ
Marmota_monax	HSQDPSGDSKLYRLHPCKSLQ
Marmota_monax_0	HSQDPSGDSKLYRLHPCKSLQ
Melospiza_melodia	HSQDPSGDTKLYRLHPCRSLQ
Mesembrinibis_cayennensis	HSQDPSGDTKLYRLHPCRSLQ
Mesitornis_unicolor	HSQDPSGDTKLYRLHPCRSLQ
Microcaecilia_unicolor	HSQDPSGDSKLYRLHPCRSLQ
Mionectes_macconnelli	HSQDPSGDTKLYRLHPCRSLQ
Molossus_molossus	HSQDPSGDSKLYRLHPCRSLQ
Molothrus_ater	HSQDPSGDTKLYRLHPCRSLQ
Monodon_monoceros	HSQDPSGDSKLYRLHPCRSLQ
Moschus_moschiferus	HSQDPSGDSKLYRLHPCRSLQ
Motacilla_alba	HSQDPSGDTKLYRLHPCRSLQ
Muntiacus_muntjak	HSQDPSGDSKLYRLHPCRSLQ
Muntiacus_reevesi	HSQDPSGDSKLYRLHPCRSLQ
Mus_caroli	HSQDPSGDSKLYRLHPCKSLQ
Mus_musculus	HSQDPSGDSKLYRLHPCKSLQ
Mus_musculus_0	HSQDPSGDSKLYRLHPCKSLQ
Mus_spicilegus	HSQDPSGDSKLYRLHPCKSLQ
Myiagra_hebetior	HSQDPSGDTKLYRLHPCRSLQ
Myotis_brandtii	HSQDPSGDSKLYRLHPCRSLQ
Myotis_davidii	HSQDPSGDSKLYRLHPCRSLQ
Myotis_lucifugus	HSQDPSGDSKLYRLHPCRSLQ
Mystacornis_crossleyi	HSQDPSGDTKLYRLHPCRSLQ
Nannospalax_galili	HSQDPSGDSKLYRLHPCKSLQ
Neomonachus_schauinslandi	HSQDPSGDSKLYRLHPCRSLQ
Neophocaena_asiaeorientalis_asiaeorientalis	HSQDPSGDSKLYRLHPCRSLQ
Neopipo_cinnamomea	HSQDPSGDTKLYRLHPCRSLQ
Neovison_vison	HSQDPSGDSKLYRLHPCRSLQ
Neovison_vison_0	HSQDPSGDSKLYRLHPCRSLQ
Nicator_chloris	HSQDPSGDTKLYQLHPCRSLQ
Nipponia_nippon	HSQDPSGDTKLYRLHPCRSLQ
Notiomystis_cincta	HSQDPSGDTKLYRLHPCRSLQ
Nyctereutes_procyonoides	HSQDPSGDSKLYRLHPCRSLQ
Nyctibius_bracteatus	HSQDPSGDTKLYRLHPCRSLQ
Nyctibius_grandis	HSQDPSGDTKLYRLHPCRSLQ
Nycticryphes_semicollaris	HSQDPSGDTKLYRLHPCRSLQ
Nyctiprogne_leucopyga	HSQDPSGDTKLYRLHPCRSLQ
Oceanites_oceanicus	HSQDPSGDTKLYRLHPCRSLQ
Oceanodroma_tethys	HSQDPSGDTKLYRLHPCRSLQ
Octodon_degus	HSQDPSGDSKLYRLHPCKSLQ
Odobenus_rosmarus_divergens	HSQDPSGDSKLYRLHPCRSLQ
Oenanthe_oenanthe	HSQDPSGDTKLYRLHPCRSLQ
Onychorhynchus_coronatus	HSQDPSGDTKLYRLHPCRSLQ
Opisthocomus_hoazin	HSQDPSGDTKLYRLNPCRSLQ
Oreocharis_arfaki	HSQDPSGDTKLYRLHPCRSLQ
Origma_solitaria	HSQDPSGDTKLYRLHPCRSLQ
Oriolus_oriolus	HSQDPSGDTKLYRLHPCRSLQ
Ornithorhynchus_anatinus	HSQDPSGDTKLYRLHPCRSLQ
Otus_sunia	HSQDPSGDTKLYRLHPCRSLQ
Ovis_aries	HSQDPSGDSKLYRLHPCRSLQ
Ovis_aries_0	HSQDPSGDSKLYRLHPCRSLQ
Oxylabes_madagascariensis	HSQDPSGDTKLYRLHPCRSLQ
Oxyruncus_cristatus	HSQDPSGDTKLYRLHPCRSLQ
Pachycephala_philippinensis	HSQDPSGDTKLYRLHPCRSLQ
Pachyramphus_minor	HSQDPSGDTKLYRLHPCRSLQ
Pan_paniscus	HSQDPSGDSKLYRLHPCRSLQ

Pan_Panthera_pardus	HSQDPSGDAKLYRLHPCRSLQ
Pandion_haliaetus	HSQDPSGDTKLYRLHPCRSLQ
Panthera_leo	HSQDPSGDAKLYRLHPCRSLQ
Panthera_tigris_altaica	HSQDPSGDAKLYRLHPCRSLQ
Panurus_biarmicus	HSQDPSGDTKLYRLHPCRSLQ
Panurus_biarmicus_0	HSQDPSGDTKLYRLHPCRSLQ
Pardalotus_punctatus	HSQDPSGDTKLYRLHPCRSLQ
Patagioenas_fasciata_monilis	HSQDPSGDTKLYRLNPCRSLQ
Patagioenas_fasciata_monilis_0	HSQDPSGDTKLYRLNPCRSLQ
Pedionomus_torquatus	HSQDPSGDTKLYRLHPCRSLQ
Pelecanoides_urinaPluvianellus_socialis	HSQDPSGDTKLYRLHPCRSLQ
Pelecanus_crispus	HSQDPSGDTKLYRLHPCRSLQ
Pelodiscus_sinensis	HSQDPSGDTKLYRLHPCRSLQ
Pelusios_castaneus	HSQDPSGDTKLYRLHPCRSLQ
Peucedramus_taeniatus	HSQDPSGDTKLYRLHPCRSLQ
Phaetusa_simplex	HSQDPSGDTKLYRLHPCRSLQ
Phainopepla_nitens	HSRDPSGDTKLYRLHPCRSLR
Phalacrocorax_carbo	HSQDPSGDTKLYRLHPCRSLQ
Phocoena_sinus	HSQDPSGDSKLYRLHPCRSLQ
Phyllostomus_discolor	HSQDPSGDSKLYRLHPCRSLQ
Pipra_filicauda	HSQDPSGDTKLYRLHPCRSLQ
Piprites_chloris	HSQDPSGDTKLYRLHPCRSLQ
Platysteira_castanea	HSQDPSGDTKLYRLHPCRSLQ
Platysternon_megacephalum	HSQDPSGDTKLYRLHPCRSLQ
Ploceus_nigricollis	HSQDPSGDTKLYRLHPCRSLQ
Podargus_sScopus_umbretta	HSQDPSGDTKLYRLHPCRSLQ
Podilymbus_podiceps	HSQDPSGDTKLYRLHPCRSLQ
Poecile_aNesospiza_acunhae	HSQDPSGDTKLYRLHPCRSLQ
Pogona_vitticeps	HSQDPSGESKLYRLHPCRSLQ
Polioptila_caerulea	HSQDPSGDTKLYRLHPCRSLQ
Pomatorhinus_ruficollis	HSQDPSGDTKLYRLHPCRSLQ
Pomatostomus_ruficeps	HSQDPSGETKLYRLHPCRSLQ
Promerops_cafer	HSQDPSGDTKLYRLHPCRSLQ
Propithecus_coquereli	HSQDPSGDSKLYRLHPCRSLQ
Prunella_fulvescens	HSQDPSGDTKLYRLHPCRSLQ
Prunella_himalayana	HSQDPSGDTKLYRLHPCRSLQ
Psophia_crepitans	HSQDPSGDTKLYRLHPCRSLQ
Pterocles_burchelli	HSQDPSGDTKLYRLHPCRSLQ
Pterocles_gutturalis	HSQDPSGDTKLYRLHPCRSLQ
Pteropus_alecto	HSQDPSGDSKLYRLHPCRSLQ
Pteropus_vampyrus	HSQDPSGDSKLYRLHPCRSLQ
Pteruthius_melanotis	HSQDPSGDTKLYRLHPCRSLQ
Ptilonorhynchus_violaceus	HSQDPSGDTKLYRLHPCRSLQ
Ptilorrhoa_leucosticta	HSQDPSGDTKLYRLHPCRSLQ
Puma_concolor	HSQDPSGDAKLYRLHPCRSLQ
Pygoscelis_adeliae	HSQDPSGDTKLYRLHPCRSLQ
Quiscalus_mexicanus	HSQDPSGDTKLYRLHPCRSLQ
Rattus_norvegicus	HSQDPSGDSKLYRLHPCKSLQ
Rhabdornis_inornatus	HSQDPSGDTKLYRLHPCRSLR
Rhadina_sibilaHypocryptadius_cinnamomeus	HSQDPSGDTKLYRLHPCRSLQ
Rhagologus_leucostigma	HSQDPSGDTKLYRLHPCRSLQ
Rhegmatorhina_hoffmannsi	HSQDPSGDTKLYRLHPCRSLQ
Rhinolophus_ferrumequinum	HSQDPSGESKLYRLHPCRSLQ
Rhipidura_dahli	HSQDPSGDTKLYRLHPCRSLQ
Rhodinocichla_rosea	HSQDPSGDTKLYRLHPCRSLQ
Rhynochetos_jubatus	HSQDPSGDTKLYRLLPCRSLQ
Rissa_Uria_aalge	HSQDPSGDTKLYRLHPCRSLQ
RosTrogon_melanurus	HSQDPSGDTKLYRLHPCRSLQ
Rousettus_aegyptiacus	HSQDPSGDSKLYRLHPCRSLQ
Rynchops_niger	HSQDPSGDTKLYRLHPCRSLQ
Sagittarius_serpentarius	HSQDPSGDTKLYRLHPCRSLQ
Sakesphorus_luctuosus	HSQDPSGDTKLYRLHPCRSLQ
Salvator_merianae	HSQDPSGESKMYRLHPCRSLQ
Sciurus_vulgaris	HSQDPSGDSKLYRLHPCKSLQ

Sclerurus_mexicanus	HSQDPSGDTKLYRLHPCRSLQ
Scytalopus_superciliaris	HSQDPSGDTKLYRLHPCRSLQ
Serinus_canaria	HSQDPSGDTKLYRLHPCRSLQ
Setophaga_kirtlandii	HSQDPSGDTKLYRLHPCRSLQ
Sitta_europaea	HSQDPSGDTKLYRLHPCRSLQ
Smutsornis_africanus	HSQDPSGDAKLYRLHPCKSLQ
SOrthonyx_spaldingii	HSQDPSGDTKLYRLHPCRSLQ
Spheniscus_mendiculus	HSQDPSGDTKLYRLHPCRSLQ
Spizaetus_tyrannus	HSQDPSGDTKLYRLHPCRSLQ
Spizella_passerina	HSQDPSGDTKLYRLHPCRSLQ
SSus_scrofa	HSQDPSGDSKLYRLHPCRSLQ
Steatornis_caripensis	HSQDPSGDTKLYRLHPCRSLQ
Stercorarius_parasiticus	HSQDPSGDTKLYRLHPCRSLQ
Sula_dactylaPicathartes_gymnocephalus	HSQDPSGDTKLYRLHPCRSLR
Suricata_suricatta	HSQDPSGDAKLYRLHPCRSLQ
Sus_scrofa	HSQDPSGDSKLYRLHPCRSLQ
Sus_scrofa_0	HSQDPSGDSKLYRLHPCRSLQ
Sylvia_aChionis_minor	HSQDPSGDTKLYRLHPCRSLQ
Syrrhaptes_paradoxus	HSQDPSGDTKLYRLHPCRSLQ
Tachuris_rubrigasRegulus_saBucorvus_abyssinicus	HSQDPSGDTKLYRLHPCRSLQ
Taeniopygia_guttata	HSQDPSGDTKLYRLHPCRSLQ
Taeniopygia_guttata_0	HSQDPSGDSKLYRLHPCRSLQ
Tauraco_erythrolophus	HSQDPSGDTKLYRLHPCRSLQ
Terrapene_carolina_Bos_mutus	HSQDPSGDSKLYRLHPCRSLQ
Thalassarche_chlororhynchos	HSQDPSGDTKLYRLHPCRSLQ
Thinocorus_orbignyianus	HSQDPSGDTKLYRLHPCKSLQ
Tichodroma_muraria	HSQDPSGDTKLYRLHPCRSLQ
Todus_mexicanus	HSQDPSGDTKLYRLHPCRSLQ
Toxostoma_redivivum	HSQDPSGDTKLYRLHPCRSLR
Tupaia_chinensis	HSQDPSGDAKLYRLHPCRSLQ
Tursiops_Physeter_macrocephalus	HSQDPSGDSKLYRLHPCRSLQ
Tychaedon_coryphoeus	HSQDPSGDTKLYRLHPCRSLQ
Tyrannus_savana	HSQDPSGDTKLYRLHPCRSLQ
Urocitellus_parryii	HSQDPSGDSKLYRLHPCKSLQ
Urocolius_indicus	HSQDPSGDTKLYRLHPCRSLQ
Urocynchramus_pylzowi	HSQDPSGDTKLYRLHPCRSLQ
Ursus_americanus	HSQDPSGDSKLYRLHPCRSLQ
Ursus_arctos_horribilis	HSQDPSGDSKLYRLHPCRSLQ
Ursus_maritimus	HSQDPSGDSKLYRLHPCRSLQ
Varanus_komodoensis	HSQDPSGESKLYRLHPCRSLQ
Vicugna_pacos	HSQDPSGDSKLYRLHPCRSLQ
Vidua_macroura	HSQDPSGDTKLYRLHPCRSLQ
Vireo_altiloquus	HSQDPSGDTKLYRLHPCRSLQ
Vulpes_vulpes	HSQDPSGDSKLYRLHPCRSLQ
Xiphorhynchus_elegans	HSQDPSGDTKLYRLHPCRSLQ
Zalophus_californianus	HSQDPSGDSKLYRLHPCRSLQ
Zapornia_aAnhinga_rufa	HSQDPSGDTKLYRLHPCRSLQ
ZonoZosterops_lateralis_melanops	HSQDPSGDTKLYRLHPCRSLQ
Zosterops_hypoxanthus	HSQDPSGDTKLYRLHPCRSLQ

Supplementary Figure S2. RMSD of 250 ns molecular dynamics simulation trajectories of Kaiso P588A and P588^{cis} complexes with KBS DNA.

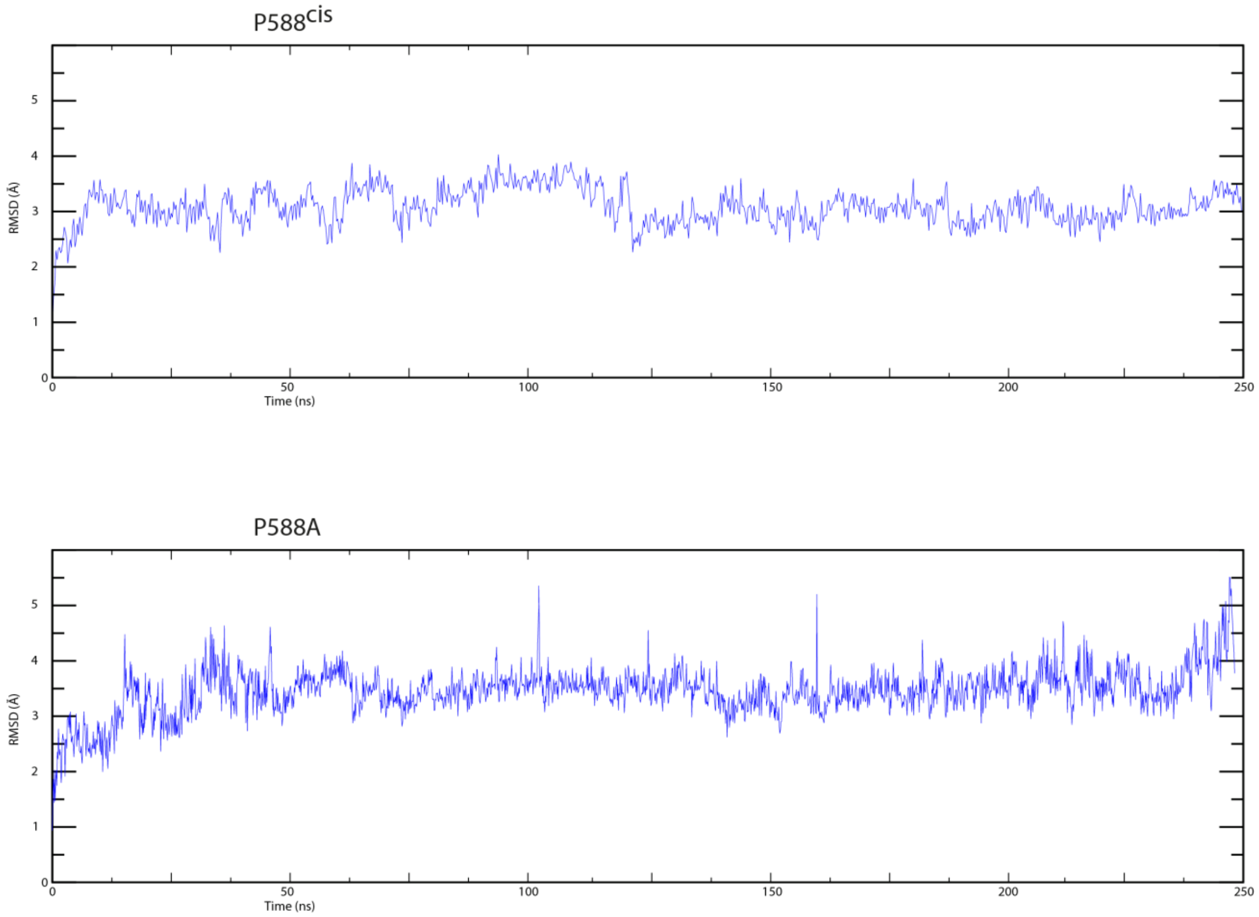

.

Supplementary Figure S3. RMSD of molecular dynamics simulation trajectories of Kaiso complex with KBS DNA.

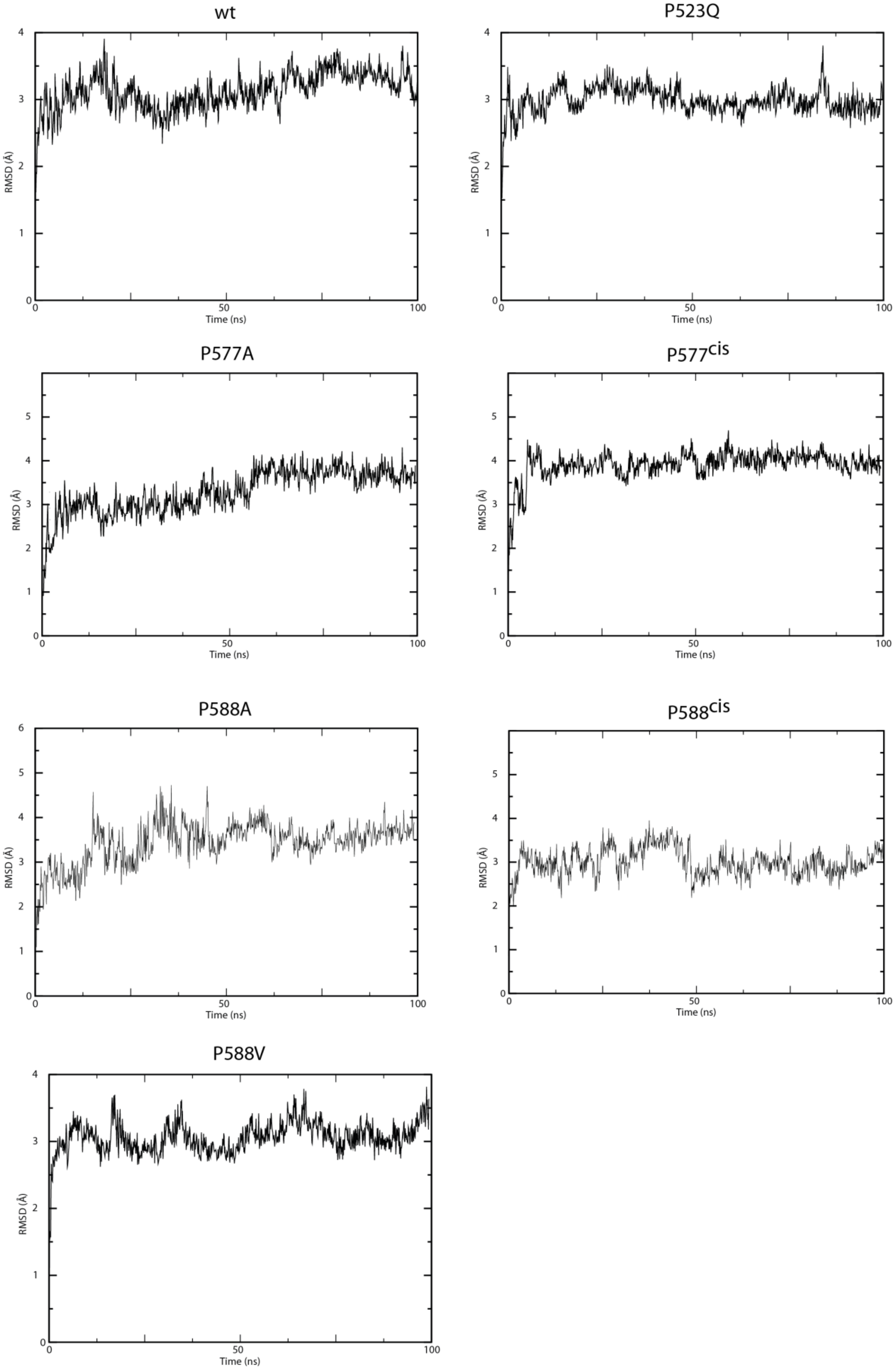

Supplementary Figure S4. RMSD of molecular dynamics simulation trajectories of Kaiso complex with methylated DNA.

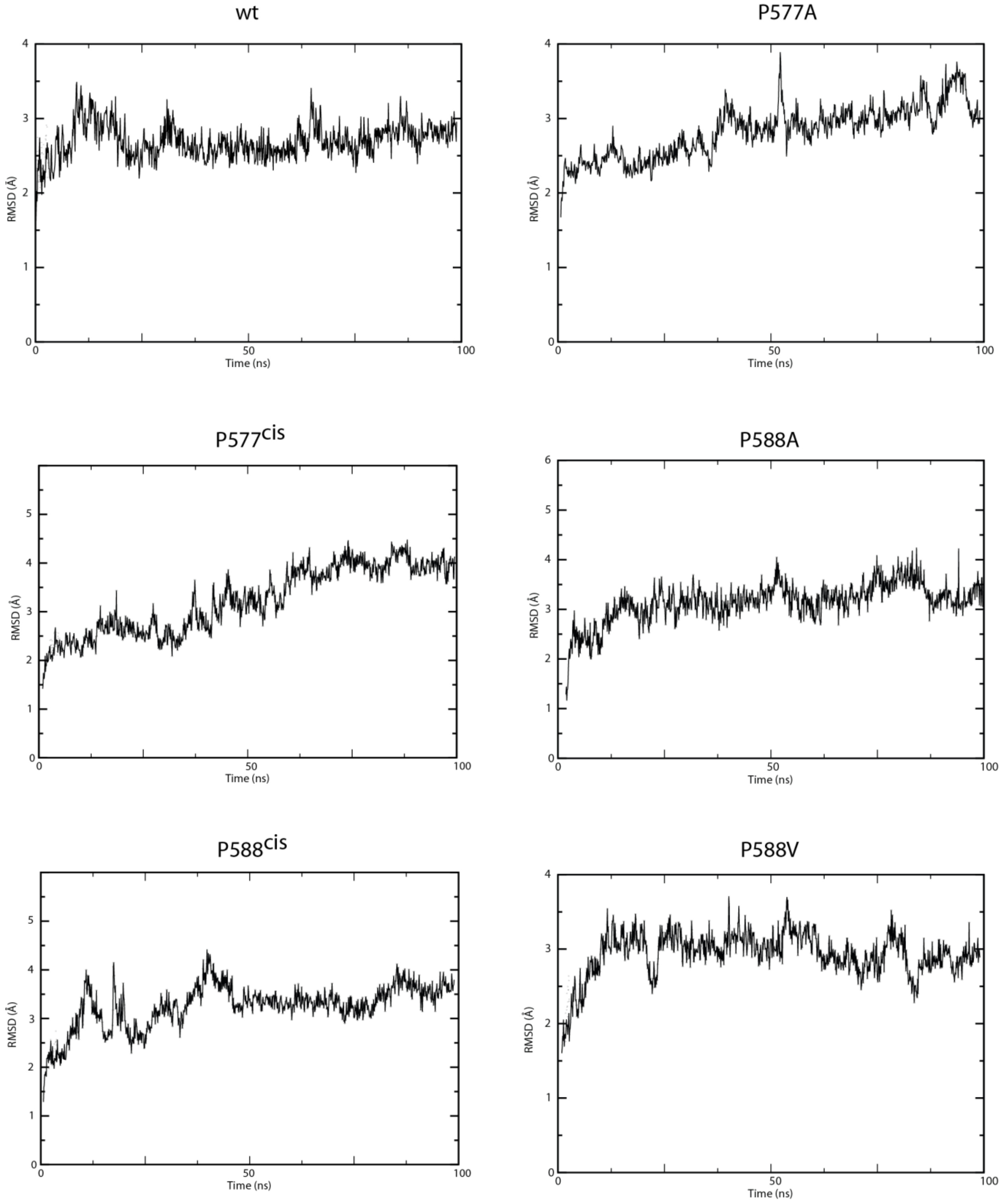

Supplementary Figure S5. (a) Protein preparations used for EMSA. (b) Non-specific binding controls at 10 μM.

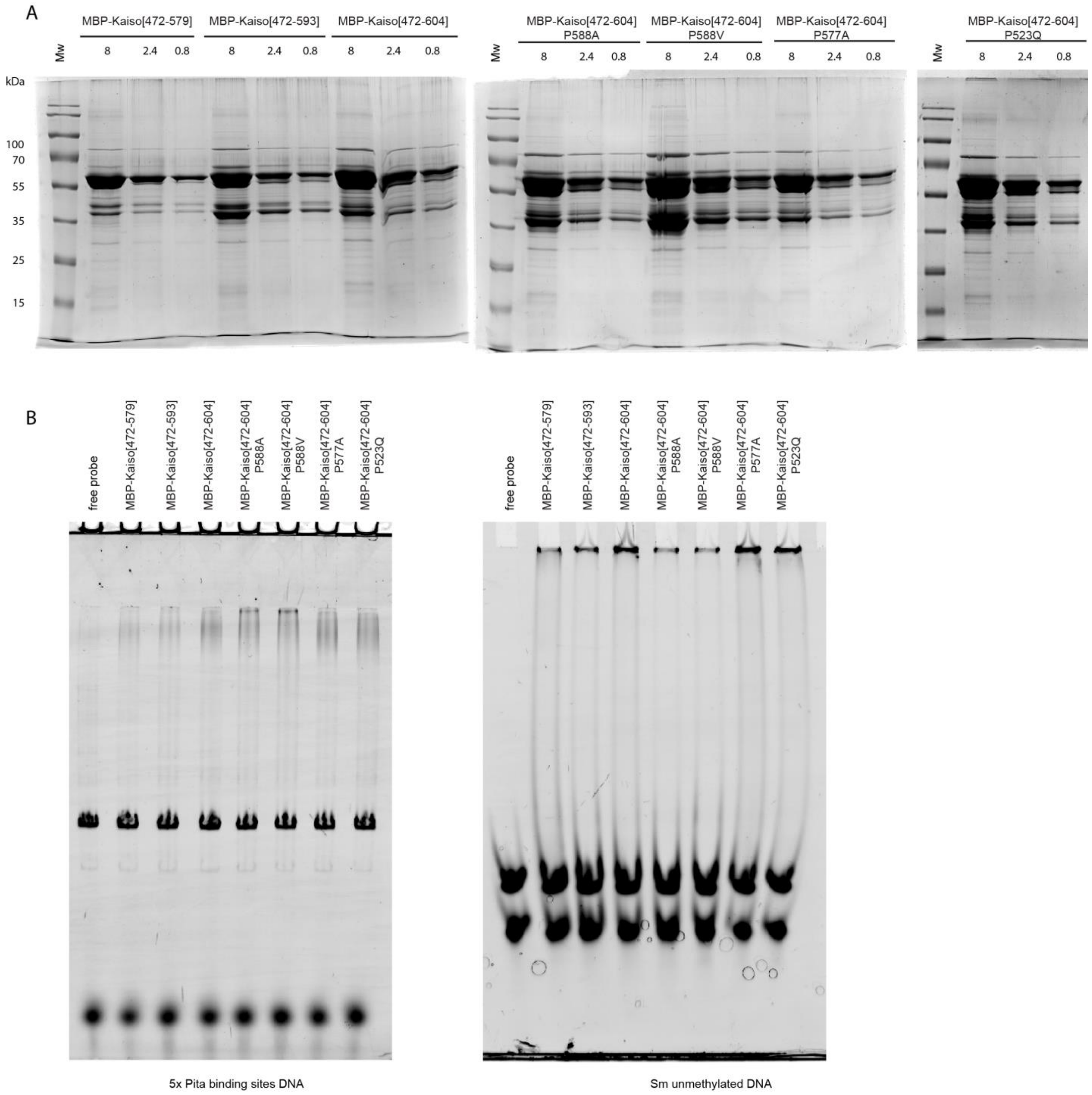

Supplementary Figure S6. Uncropped gels of EMSA.

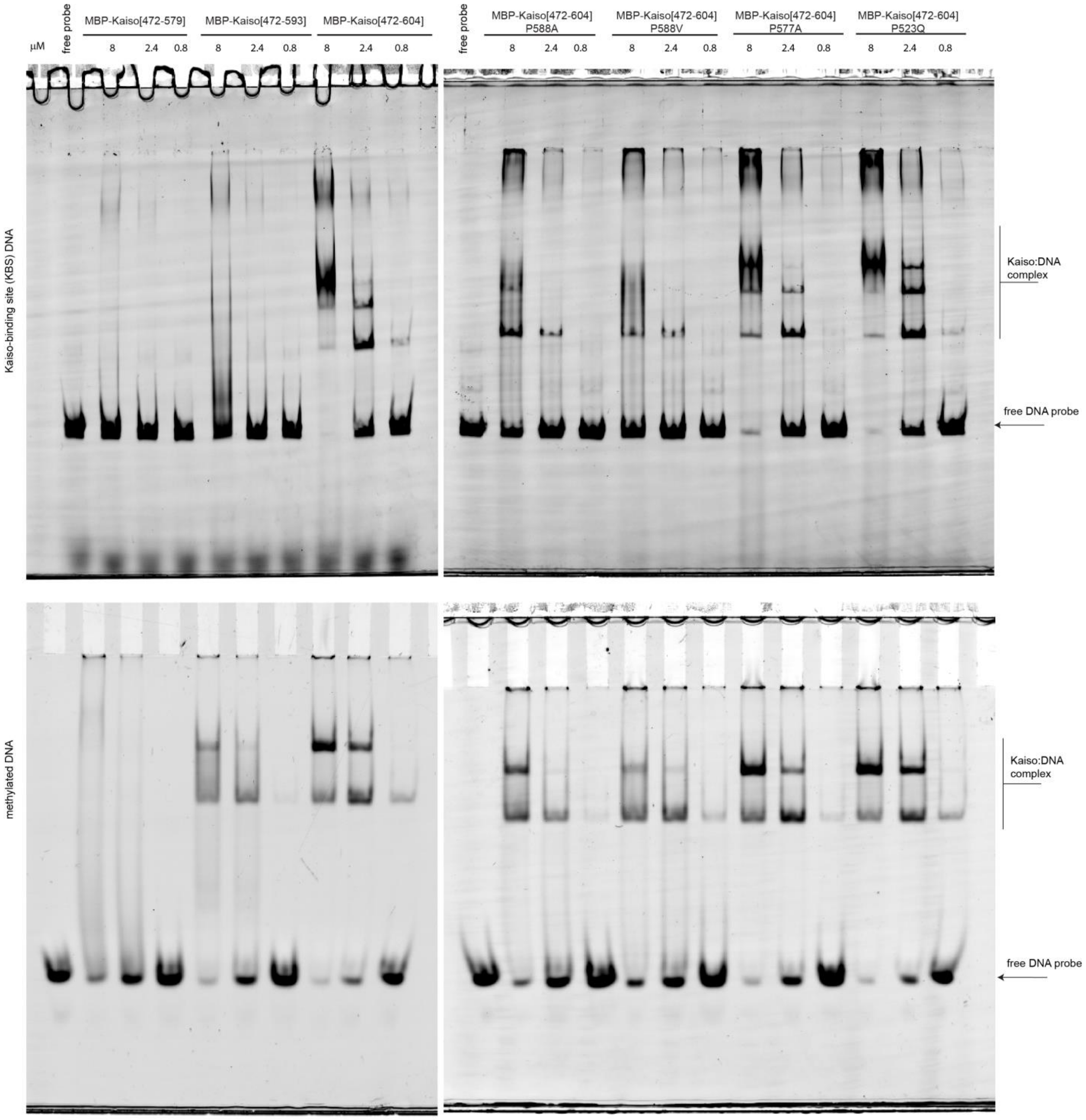

Supplement: Supplementary file 1 [file ijms-23-15494-s001.zip › ijms-2021907-supplementary.pdf]
